# Castration of Dogs Using Local Anesthesia After Sedating With Xylazine and Subanesthetic Doses of Ketamine

**DOI:** 10.3389/fvets.2019.00478

**Published:** 2020-01-23

**Authors:** Erika Silva, John Schumacher, Thomas Passler

**Affiliations:** ^1^Facultad de Ciencias Clinicas Veterinarias, Instituto de Ciencias Clinicas Veterinarias, Universidad Austral de Chile, Valdivia, Chile; ^2^Department of Clinical Sciences, College of Veterinary Medicine, Auburn University, Auburn, AL, United States

**Keywords:** dog, castration, local anesthetic, xylazine, subanesthetic ketamine

## Abstract

Orchiectomy is performed in some species using only sedation and local anesthesia to decrease the expense of performing the procedure using general anesthesia. The objective of this study was to determine if dogs can be castrated safely and painlessly by using only sedation and local anesthesia. After dogs were sedated with intramuscularly administered xylazine (1 mg/kg) and subanesthetic ketamine (1 mg/kg), testes and skin were anesthetized with 2% lidocaine (6 mg/kg, total dose) buffered with sodium bicarbonate. Systolic, diastolic, and mean blood pressures; heart and respiratory rates; and movement scores were determined before and during surgery when manipulations were most likely to cause pain. No dog reacted substantially to injection of the combination of sedatives, and no dog reacted noticeably to injection of lidocaine. During surgery, the average heart rate was reduced from baseline by 40–60 beats per minute, and the average respiratory rate was reduced by ~10 breaths per minute. An overall reduction of arterial blood pressures was observed. All but one dog moved purposely in response to a toe pinch at the end of surgery. We found that sedating dogs intramuscularly with xylazine and a sub-anesthetic dose of ketamine and administering lidocaine at the incision site and intratesticularly allowed dogs to be castrated humanely and avoided the expense of general anesthesia and the need for hospitalization.

## Introduction

When anesthetic equipment is not available or large numbers of dogs must be castrated, chemical immobilization in conjunction with local anesthesia may be an economical and expeditious method of performing the procedure.

Orchiectomy in other species can be performed painlessly after injecting the testes or spermatic cords with local anesthetic. Men can be castrated using local anesthesia, often without sedation (1–4). Stallions also are often castrated while they are standing by using only sedation, local anesthesia, and a lip twitch for restraint (5).

In a study that evaluated the analgesic effect of intratesticular and incision line infiltration of local anesthetic (0.5% ropivacaine) (6), 7 of 11 sedated dogs were castrated with little or no movement. Because of substantial movement, four dogs in that study were given general anesthesia to complete the castration; however, the authors did not offer an opinion as to whether movement appeared to be in response to surgically induced pain or other environmental stimuli. Silva Cuzmar et al. (7) investigated the use of sedation and local anesthesia for immobilizing dogs during castration. That study demonstrated that dogs can be castrated without causing signs of pain by administering xylazine for restraint, followed by administering lidocaine intratesticularly and subcutaneously at the site of incision. In that study, dogs did not display signs of pain as indicated by changes in vital signs and the lack of purposeful movement in the presence of surgical stimulation. We sought to bolster that study with another study in which blood pressure was monitored before and during surgery. Blood pressure has been shown to be a more sensitive indicator of nociception than heart rate (HR) during castration of stallions (8) or ovariohysterectomy of dogs (9). We also wanted to know if lidocaine administered at a standardized dose much less than the recommended maximum therapeutic dose for the dog [10 mg/kg (10)] would allow castration without evidence of pain. Because injection of lidocaine can be painful (11, 12), we also wanted to determine the effect of using buffered lidocaine for preventing signs of pain during injection of testes and the incision site. In our previous study (7), non-purposeful movement of dogs during surgery was common. We wondered if a sedative dose of ketamine in addition to xylazine HCL would decrease movement. For this study, we recorded time of administration of local anesthesia and start of surgery, information that would indicate the necessary time to wait before administering local anesthesia and expectation that the testes and skin are desensitized.

## Materials and Methods

### Preparation of Dogs for Surgery

Twelve male dogs of various breeds underwent castration after owners signed a consent form allowing participation of their dogs in the study. This study was approved by the Universidad Austral de Chile Bioethics Committee for the Use of Animals in Biomedical Research (protocol No. 231/2015). Physical examinations were performed before the dogs were sedated. Dogs had been fasted overnight but were allowed access to water until they were sedated with xylazine HCl (1.0 mg/kg; Xilacina 2%, Centrovet, Santiago, Chile) and ketamine (1.0 mg/kg; Ketamina, Richmond Laboratories, Buenos Aires, Argentina) mixed in the same syringe and administered intramuscularly in the semitendinosus or semimembranosus muscle. Once recumbent, dogs were placed on a surgical table in left or right lateral recumbency and prepared for surgery with the upper pelvic limb flexed and elevated. After the surgical site was clipped of hair and scrubbed, lidocaine (6 mg/kg) was administered into the testes and site of cutaneous incision.

### Physiological Values Assessed Before and During Surgery

Systolic (SAP), diastolic (DAP), and mean (MAP) blood pressures; heart and respiratory rates; and movement scores were determined at various time points ([1 through 12]) throughout the procedure. Heart and respiratory rates and SAP, DAP, and MAP were measured using a Data Scope Trio Patient Monitor (Datascope Corporation, Mahwah, NJ, USA) and a human pediatric blood pressure cuff (7.2–13 cm) (Tuff Cuff, Cas Medical Systems Inc., Branford, CT USA) applied to the base of the tail. For dogs without a tail, blood pressures were measured by attaching the cuff to a forelimb, slightly distal to the elbow. These values were determined with the dogs standing, before they were injected with sedatives [1], and thereafter, with the dogs in lateral recumbency, 3 min after administration of the sedatives [2], immediately prior to administering lidocaine [3], and during injection of lidocaine at each of three injection sites [4–6]. The same data were collected during the cutaneous incision [7], at the time of removal of each testis [8, 9], during closure of the wound [10, 11] and after pinching interdigital skin on a pelvic limb with a Halstead forceps after the last suture was placed [12]. Four assistants (two veterinarians and two veterinary students) collected these data. Each assistant was assigned to determine one of the following indicators of nociception: change in arterial blood pressures, respiratory rates, heart rates and movement. The assistants were assigned the same task for each castration. Data collected immediately before administering the sedatives were used as baseline [1]. Movement was subjectively graded on a scale of 0–3 where: 0 = no movement; 1 = slight to moderate, non-purposeful movement that did not interfere with surgical manipulations; 2 = purposeful movement that interfered substantially with surgery; and 3 = purposeful movement that made continuation of surgery impossible.

### Administration of Local Anesthetic

Lidocaine HCl (Lidocalm^®^ 2%, Drag Pharma, Santiago, Chile) diluted with 8.4% sodium bicarbonate (Laboratorio Sanderson S.A., San Joaquin, Chile) as a 9:1 ratio was divided into three equal doses and administered by the first author into each testis, and then, subcutaneously, on the midline, cranially to the scrotum. Testicular injections were made by inserting a 25-gauge, 16-mm needle at the median raphe and then directing the needle, from its subcutaneous location into the parenchyma of each testis without withdrawing the needle through the skin between injections. The time from the subcutaneous, prescrotal injection of lidocaine to the skin incision was recorded.

### Surgical Procedure

Open castrations were performed using a prescrotal approach with the dog in lateral recumbency and its upper pelvic limb elevated in a flexed position. The pampiniform plexus and vas deferens were ligated together using a transfixion ligature of 2/0 or 3/0 polyglycolic acid (Safil, B. Braun Surgical S.A., Rubí, Spain). Subcutaneous tissue was closed with the same suture using a simple-continuous pattern. Skin was closed with non-absorbable 3/0 synthetic monofilament suture (Dafilon, polyamide, B. Braun Medical S.A., Bogotá, Colombia) using a simple-interrupted pattern. The first author performed all surgeries. All dogs were administered a single dose of ketoprofen (1 mg/kg IM) (KET-10, Drag Pharma Chile Invetec S.A., Lautaro 300, Quilicura, Santiago, Chile) and procaine penicillin G (20,000 IU/kg IM) after surgery. Time from placing the last skin suture until the dog was standing was recorded. Dogs that remained recumbent after the toe pinch were placed on a pad on the surgery room floor in lateral recumbency. The dogs were discharged between 4 and 6 h after they recovered from sedation. Owners were contacted by telephone 4–5 days after surgery for their opinion of their dog's health.

### Data Analyses

Statistical analyses were performed using a commercially available software package (JMP^®^ 13.0.0, SAS Institute Inc., Cary, NC, USA). Data were assessed for normality by visual inspection of frequency distributions and by the Shapiro–Wilk test. Descriptive statistics, including mean, median, standard deviation, minimum, and maximum were calculated. Statistical tests were performed to test the null hypothesis that the means of each outcome variable assessing pain were equal at each time point during surgery. Normally distributed outcome variables (heart rate, respiratory rate, and arterial blood pressures) were analyzed using a mixed model analysis for repeated measures. Model fit was assessed by visual inspection of residual plots and comparison of the Akaike information criterion (AIC) for each model. *Post-hoc* multiple comparisons were performed using the Dunnett's test to compare each time point with baseline (time point 1). Movement scores were not normally distributed; thus, the Friedman's test, a non-parametric alternative for the repeated measures ANOVA, was used. The Steel method, a non-parametric alternative for the Dunnett's test, was used for multiple comparisons of movement scores with baseline. A value of *p* < 0.05 was considered significant.

## Results

**Figure 1:**
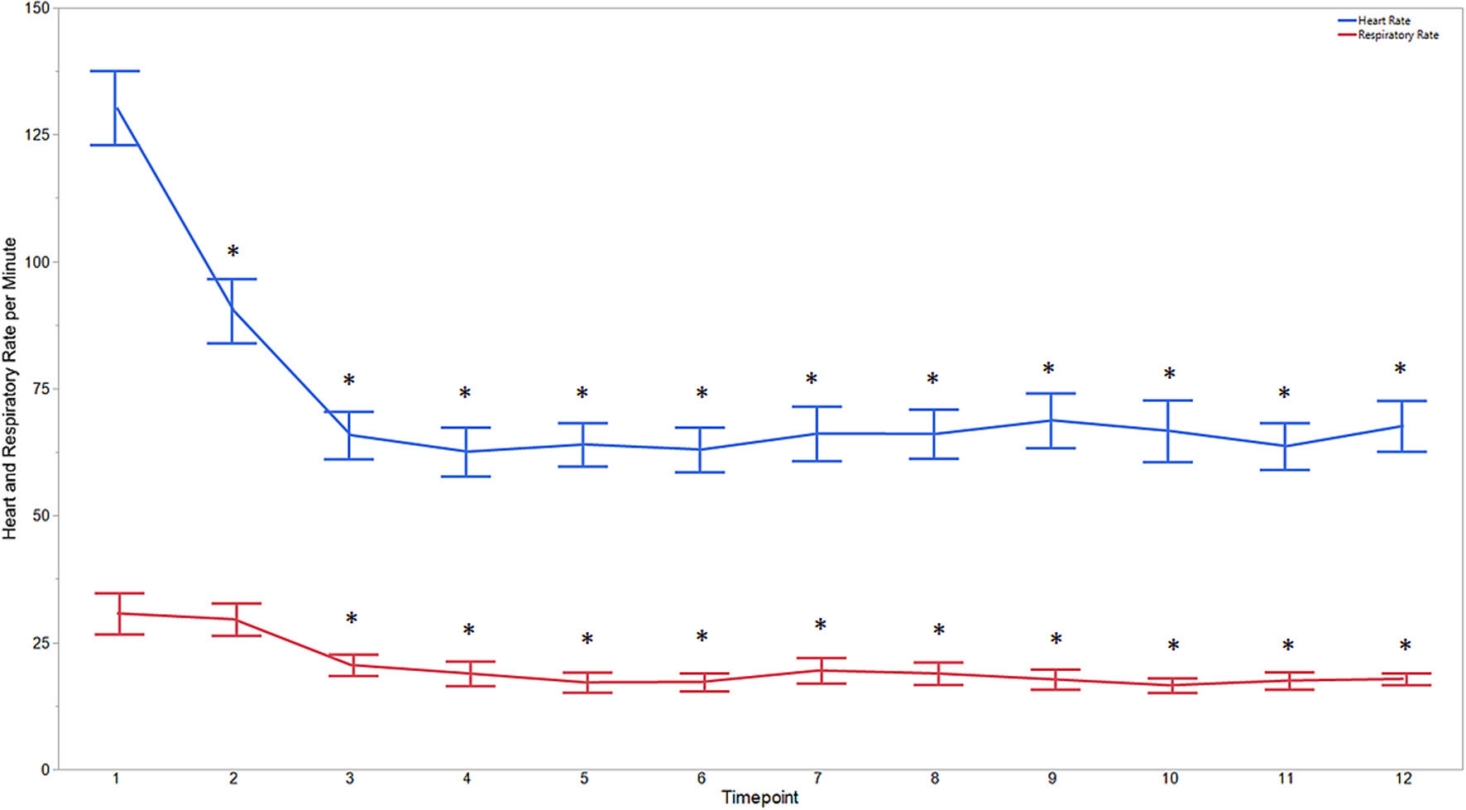
Average heart and respiratory rates at 12 time points of dogs castrated while sedated with xylazine and a subanesthetic dose of ketamine. Time points are: 1 = baseline, before any drugs, 2 = 3 min post xylazine/ketamine, 3 = before testicle injection, 4 = during first testicle injection, 5 = during second testicle injection, 6 = during skin injection, 7 = during incision, 8 = during first testicle removal, 9 = during second testicle removal, 10 = during subcutaneous suture, 11 = during skin suture, 12 = after toe pinch. ^*^Reflects significant difference to baseline (time point 1). Error bars were constructed using the SEM.

**Figure 2:**
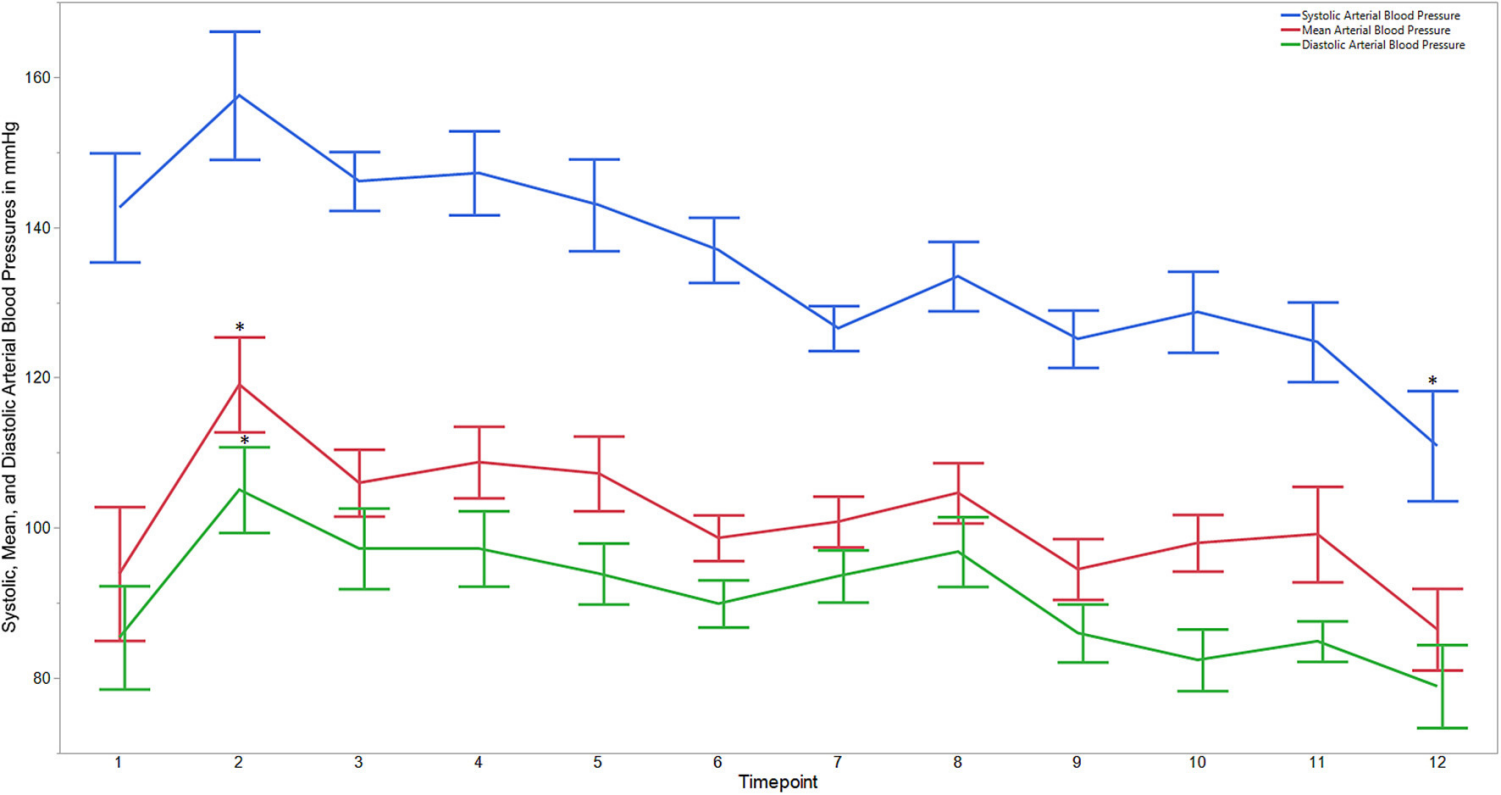
Average systolic, mean, and diastolic arterial blood pressures for dogs castrated while sedated with xylazine and a subanesthetic dose of ketamine. Time points are: 1 = baseline, before any drugs, 2 = 3 min post xylazine/ketamine, 3 = before testicle injection, 4 = during first testicle injection, 5 = during second testicle injection, 6 = during skin injection, 7 = during incision, 8 = during first testicle removal, 9 = during second testicle removal, 10 = during subcutaneous suture, 11 = during skin suture, 12 = after toe pinch. ^*^Reflects significant difference to baseline (time point 1). Error bars were constructed using the SEM.

**Figure 3:**
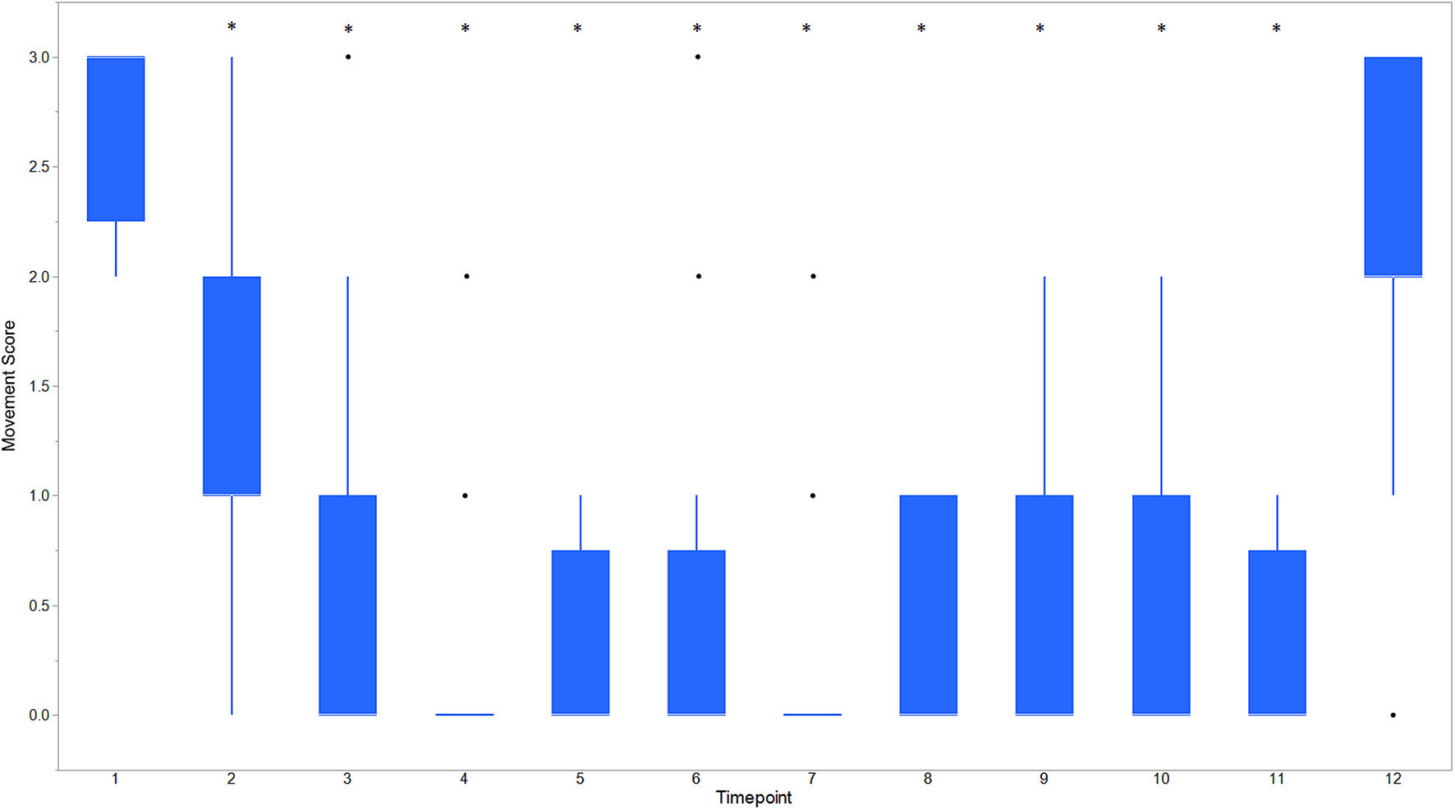
Boxplot of movement scores for dogs castrated while sedated with xylazine and a subanesthetic dose of ketamine. Time points are: 1 = baseline, before any drugs, 2 = 3 min post xylazine/ketamine, 3 = before testicle injection, 4 = during first testicle injection, 5 = during second testicle injection, 6 = during skin injection, 7 = during incision, 8 = during first testicle removal, 9 = during second testicle removal, 10 = during subcutaneous suture, 11 = during skin suture, 12 = after toe pinch. ^*^Reflects significant difference to baseline (time point 1) movement was subjectively graded on a scale of 0–3 where: 0 = no movement; 1 = slight to moderate, non-purposeful movement that did not interfere with surgical manipulations; 2 = purposeful movement that interfered substantially with surgery; and 3 = purposeful movement that made continuation of surgery impossible. ^*^Reflects significant difference to baseline (time point 1). Individual black dots reflect outliers.

Descriptive statistics for age and weight of dogs and lengths of different phases of the castration procedure are displayed in Table 1. No dog reacted substantially to injection of the combination of sedatives, and no dog reacted noticeably to injection of lidocaine. During surgery, the average heart rate was reduced from baseline by 40–60 beats per minute, and the average respiratory rate was reduced by ~10 breaths per minute. The reduction in each of these rates from baseline values was statistically significant (*p*_time_ < 0.0001) (Figure 1). With the exception of time point 2 (3 min after administration of the sedatives), an overall reduction of arterial blood pressures were observed. The SAP, DAP, and MAP varied significantly over time (*p*_time_ < 0.05), but each outcome variable was significantly different from baseline only at one time point during surgery (*p*_diff_ = 0.0011, 0.0122, and 0.0103, respectively) (Figure 2). During surgery, movement scores were significantly lower than at baseline (*p* < 0.0093) at all time points, with the exception of time-point 12 (the time at which the toe pinch test was applied at the end of surgery), during which movement scores were similar to those at baseline (*p* = 0.2262) (see Figure 3). Four dogs stood in response to the toe pinch. Complications associated with the surgery other than slight swelling and redness of the incision site, were not reported by owners who were contacted by telephone at 4 or 5 days after their dog's castration. Hospital staff, who removed sutures 9–12 days after surgery, reported no owner complaints associated with the surgery.

**Table 1:** Age and weight of castrated dogs, and time measurements of different phases of the castration procedure are summarized in this table.

Age (months)	Median	9
	SD	17
	Range	7–60
Weight (kg)	Mean	21.6
	SD	8.2
	Range	9.0–33.2
Minutes from injection of sedatives to injection of lidocaine	Mean SD	17.5 7.6
	Range	5.0–33.0
Duration of time during injection of lidocaine	Median	3.0
	SD	0.8
	Range	1.0–4.0
Minutes between injection of lidocaine to skin incision	Median SD	2.0 0.9
	Range	0.0–3.0
Surgery time (min) from incision to placement of the last suture	Mean SD	12.3 4.2
	Range	8.0–22.0
Minutes from placement of the last suture to standing position (likely influenced by the application of a toe pinch).	Median SD Range	2.0 5.3 0.0–14.0

*The mean is displayed for normally distributed outcome variables, while the median was included as the measure of central tendency for non-normal data*.

## Discussion

The physiological effects of noxious stimulation include increased heart and respiratory rates and blood pressure. Heart and respiratory rates and blood pressure did not increase during surgical manipulations in this study. These autonomic responses are modulated in the brain stem in response to noxious stimulation and are not necessarily evidence of pain, because nociception does not become interpreted as pain until noxious signals reach the cerebral cortex (13). Lack of an autonomic response to noxious stimulation is evidence that nociceptive inputs never reached the spinal tracts that carry nociceptive signals to the brain, where they could be interpreted as pain. Dogs moved not at all or only so minimally during surgery that the movement did not interfere with the surgical procedure. Most dogs (11 of 12) responded to a toe pinch applied at the end of surgery with purposeful movement, with some (*n* = 4) attaining a standing position, indicating cognitive perception of a noxious stimulus, not present during castration. Purposeful movement in response to the toe pinch test indicates that lack of response to surgical manipulations was due to the effects of lidocaine, rather than the analgesic effects of xylazine and the sub-anesthetic dose of ketamine. These findings support findings of the previous study that concluded dogs likely experience no pain during castration performed using only sedation and local anesthesia (7). In that study, dogs castrated using only sedation and local anesthesia showed no purposeful movement and no increase in heart or respiratory rate during the procedure.

In this study, we found that surgery was possible soon after administration of local anesthetic with a median time of only 2 min between administration of lidocaine and commencement of surgery. In men, rapid onset of anesthesia of the testis after intratesticular injection of lidocaine was demonstrated, as painless testicular biopsy was possible when performed within 15 s after injection of lidocaine in scrotal skin and directly into the testicle (14).

Xylazine was chosen for sedation in the previous study of dog castrations performed with local anesthesia (7), because one of the aims of that study was to demonstrate an economical method of dog castration, and because other alpha2-adrenoreceptor agonists are not available in some countries. For the current study, a sub-anesthetic dose of ketamine was added to the sedation protocol to decrease the possibility of the dogs experiencing pain during intra-testicular injection of local anesthetic solution. Sodium bicarbonate was added to lidocaine for intratesticular injection of local anesthetic solution for the same reason, because lidocaine and other local anesthetic agents have a low pH, which may result a burning sensation during injection. Adding bicarbonate to the local anesthetic solution may decrease pain by raising the pH of the solution (11, 12). The sodium bicarbonate added to local anesthetic solution also increases the potency and the speed of onset of the local anesthetic solution (12, 15, 16). Pain experienced during intratesticular injection of local anesthetic solution might also be caused by rapid expansion of tissue; for this reason, injections were made with a small-bore needle to slow the rate of injection and to decrease pain associated with insertion of a needle.

Ketamine is often used at sub-anesthetic doses as a sedative in human patients. Ketamine administered at a sub-anesthetic dose appears to be particularly useful in emergency rooms for dealing with agitated patients and children, because its onset of action is about 5 min even when administered intramuscularly (17, 18), and because the incidence of side effects associated with its use is low (18–21). We administered ketamine at a fraction of the dose used in dogs (11 mg/kg) when combined with xylazine etc. (22), which would exert its effect by disconnecting the thalamocortical and limbic systems, effectively dissociating the central nervous system from outside stimuli to act as a potent analgesic permitting medical procedures to be performed without causing pain to the patient (21). Ketamine administered to human patients at a sub-anesthetic dose is also a potent analgesic (23, 24). Sub-anesthetic doses of ketamine do not significantly affect consciousness or arousal, and have no measurable cardiovascular or respiratory effects (25, 26).

The study has a number of limitations. Only young healthy dogs were studied, and the effects of the drug combination may be different in older or debilitated dogs. Although we monitored the basic aspects of cardiopulmonary function in this study, monitoring blood gases and cardiac output would have been ideal, so that the safety of the drug combinations used could have been better assessed. This was not possible, however, due to the circumstances of the study. Simultaneous administration of xylazine and ketamine to dogs, especial at high doses, can produce substantial hypoxemia (27); however, it is unlikely to be the case in the dogs of this study as they were not in a deep plane of surgical anesthesia. Emesis is sometimes a side-effect of administering xylazine to dogs (28); however, in the authors' experience emesis is unlikely to occur when xylazine is administered in association with ketamine. Nevertheless, the study did not evaluate dogs to determine if they maintained a swallow reflex after sedation, which would have been an indication that the dogs could protect their airway in the event of emesis. Although no substantial reaction was observed during intratesticular administration of local anesthetic or during the surgical procedure in this study, there may be risk that some dogs may have an aggressive reaction toward personnel during these procedures.

We found that sedating dogs intramuscularly with xylazine and a sub-anesthetic dose of ketamine and administering lidocaine at the incision site and intratesticularly allowed dogs to be castrated humanely and avoided the expense of general anesthesia.

## Data Availability Statement

The datasets generated for this study are available on request to the corresponding author.

## Ethics Statement

The animal study was reviewed and approved by Universidad Austral de Chile Bioethics Committee for the Use of Animals in Biomedical Research. Written informed consent was obtained from the owners for the participation of their animals in this study.

## Author Contributions

ES contributed to case recruitment and study execution. All authors contributed to study design and manuscript preparation. TP performed data analysis.

### Conflict of Interest

The authors declare that the research was conducted in the absence of any commercial or financial relationships that could be construed as a potential conflict of interest.
